# Homogeneous clusters of Alzheimer’s disease patient population

**DOI:** 10.1186/s12938-016-0183-0

**Published:** 2016-07-15

**Authors:** Dragan Gamberger, Bernard Ženko, Alexis Mitelpunkt, Nada Lavrač, The Alzheimer’s Disease Neuroimaging Initiative

**Affiliations:** 1 Rudjer Bošković Institute, Bijenička 54, 10000 Zagreb, Croatia; 2 Jožef Stefan Institute, Ljubljana, Slovenia; 3 Tel Aviv University, Tel Aviv, Israel; 4 University of Nova Gorica, Nova Gorica, Slovenia

**Keywords:** Alzheimer’s disease, Clustering, Biomarker identification, Brain injuries

## Abstract

**Background:**

Identification of biomarkers for the Alzheimer’s disease (AD) is a challenge and a very difficult task both for medical research and data analysis.

**Methods:**

We applied a novel clustering tool with the goal to identify subpopulations of the AD patients that are homogeneous in respect of available clinical as well as in respect of biological descriptors.

**Results:**

The main result is identification of three clusters of patients with significant problems with dementia. The evaluation of properties of these clusters demonstrates that brain atrophy is the main driving force of dementia. The unexpected result is that the largest subpopulation that has very significant problems with dementia has besides mild signs of brain atrophy also large ventricular, intracerebral and whole brain volumes. Due to the fact that ventricular enlargement may be a consequence of brain injuries and that a large majority of patients in this subpopulation are males, a potential hypothesis is that such medical status is a consequence of a combination of previous traumatic events and degenerative processes.

**Conclusions:**

The results may have substantial consequences for medical research and clinical trial design. The clustering methodology used in this study may be interesting also for other medical and biological domains.

## Background

Identification of connections between biological and clinical characteristics of Alzheimer’s disease (AD) patients is a long term goal that could significantly improve the understanding of the AD pathophysiology, improve clinical trial design, and help in predicting outcomes of mild cognitive impairment [1]. The difficulty of the task is connected to the fact that AD is clinically described as a set of signs and symptoms that can only be measured indirectly and have been integrated into various scoring systems like clinical dementia rating sum of boxes, Alzheimer’s disease assessment scale, or montreal cognitive assessment [2]. All of these scores as well as symptoms of everyday cognition problems have proved their usefulness in the diagnostic process but a unique and reliable measure or indicator does not exist. On the other side, although relations between some biological descriptors and AD diagnosis have been undoubtedly demonstrated [3, 4], currently known biological descriptors are non-specific (e.g., hippocampal volume) and their changes may be a consequence of various physiological processes. The potentially useful information related to biological causes of the cognitive status of a patient is therefore hidden in the large ‘noise’ of interfering physiological processes.

From the data analysis point of view we are searching for relevant relations in a very noisy data domain (biological descriptors) in which the target function is defined by a large set of imprecise and sometimes biased values (clinical descriptors). A simplified approach in which medical AD diagnosis is used as the target function has enabled detection of some relations, like the correlation of the decreased FDG-PET values and the AD diagnosis, but all the detected relations including those obtained by complex supervised approaches [5] have low predictive power and did not significantly improve our understanding of the disease. In line with the approach proposed in [6], our work employs a novel clustering algorithm, and aims at finding homogeneous subpopulations of AD patients in which it will be easier to identify potentially relevant relations between clinical and biological descriptors.

Clustering is a well-established machine learning methodology aimed at grouping of examples (patients) so that members in the same cluster are as similar as possible [7]. The applications are numerous and mainly aimed at the discovery of underlying concepts present in data. Popular approaches to clustering are based on distance minimization (centroid-based, k-means and hierarchical approaches) and detection of dense sets of examples (DBSCAN algorithm [8]). Distribution based clustering (expectation-maximization algorithm [9]) searches for a combination of distribution functions that can generate observed data. It presents a theoretical generalization of the distance minimization approach. On the other side, subspace clustering (CLIQUE algorithm [10]) is a type of density based approach that can be applied to domains with a large number of attributes. Its distinctive property is automatic generation of human interpretable descriptions of identified clusters.

The idea is extended in the redescription mining approach [11] where the necessary condition for the recognition of a cluster is not the density of examples but the existence of descriptions in different attribute subspaces that describe examples in the cluster. But irrespective of the implemented approach, clustering requires that important parameters like the number of clusters to be constructed, their minimal size, or maximal acceptable distance between examples are specified by the user. Often many experiments and previous experience are needed to select good parameters for a concrete application. By selecting the parameters and the way the input data are prepared the user can significantly influence the final result. The fact can be regarded as a deficiency of the methodology but also as its property that enables that available expert or background knowledge is included into the clustering process [12]. Especially problematic are domains in which examples are described by a combination of numerical and categorical attributes, because it is difficult to define appropriate distance measures, and domains that have many attributes, because the sensitivity of distance measures degrades as the number of non-informative attributes increases.

A distinguishing property of the approach presented in this work is the assumption that members of each cluster must have a common relation among a subset of attribute values. For example, if we want to identify a cluster of grown-up males from a larger set of people we can do that by detecting a set of persons that all have properties like high weight, deep voice, broad shoulders and strong arms. In order words, co-existence of potentially many properties is a good motivation to cluster examples together. The approach is different from density based algorithms because there is no requirement on actual number of examples with some property and different from redescription mining because no descriptions are generated. The resulting clusters may be similar to those obtained by distance based approaches because it can be assumed that distance between examples with co-existing properties will be small. The methodology can be regarded as an upgrade of the distance based approach in which co-existng attribute values are selected as those that are actually relevant for the computation of distances between examples. The practical implementation consists of two steps. In the first step similarity between all pairs of examples is determined through an application of a supervised learning algorithm [13]. In the second step, which is novel, possible reduction of the variability of similarity values is used to identify homogeneous clusters in single-layer and multi-layer settings [14]. Multi-layer approach is a way to ensure high homogeneity of constructed clusters. By using clinical data in one layer and biological data in the other layer and by constructing clusters that are at the same time homogeneous in both layers, the methodology seems tailored for discovery of relations that exist between clinical and biological characteristics of patients. In this work we present modification the methodology originally published in [14] and extension of the results published in [15, 16].

## Methods

Data were obtained from the Alzheimer’s disease neuroimaging initiative (ADNI) database. ADNI is a long term project aimed at the identification of biomarkers of the disease and understanding of the related pathophysiology processes. The project collects a broad range of clinical and biological data about patients with different cognitive impairment. The ADNI was launched in 2003 by the National Institute on Aging (NIA), the National Institute of Biomedical Imaging and Bioengineering (NIBIB), the Food and Drug Administration (FDA), private pharmaceutical companies and non-profit organizations. More information can be found at http://www.adni-info.org and http://www.adni.loni.usc.edu.

### ADNI dataset used in the experiments

Our work started from a set of numerical descriptors extracted from the ADNIMERGE table, the joined dataset from several ADNI data tables. We used baseline evaluation data for 916 patients in total with five different medical diagnoses: cognitive normal CN (187 patients), significant memory concern SMC (106), early mild cognitive impairment EMCI (311), late mild cognitive impairment LMCI (164), and Alzheimer’s disease AD (148). The patients are described by a total of 10 biological and 23 clinical descriptors. Biological descriptors are genetic variations of APOE4 related gene, PET imaging results FDG-PET and AV45, and MRI volumetric data of: ventricles, hippocampus, wholeBrain, entorhinal, fusiform gyrus, middle temporal gyrus (midtemp), and intracerebral volume (ICV). Clinical descriptors are: clinical dementia rating sum of boxes (CDRSB), Alzheimer’s disease assessment scale (ADAS13), mini mental state examination (MMSE), rey auditory verbal learning test (RAVLT immediate, learning, forgetting, percentage of forgetting), functional assessment questionnaire (FAQ), montreal cognitive assessment (MOCA) and everyday cognition which are cognitive functions questionnaire filled by the patient (ECogPt) and the patient study partner (ECogSP) (memory, language, visuospatial abilities, planning, organization, divided attention, and total score).

The clustering process started from one table with biological data consisting of 916 rows and 10 columns and one table with clinical data consisting of 916 rows and 23 columns. Each of the two tables represented one attribute layer. The information about medical diagnoses of the patients have not been included into the tables with the intention to use it only in the evaluation of the clustering results. The goal of the clustering process was to identify groups of patients that are large as possible and as similar as possible in terms of both layers, i.e., biological and clinical characteristics.

### Overview of the multi-layer clustering approach

Before explaining the multi-layer clustering approach, let us first assume a basic clustering task in which we have only one layer of attributes. The clustering process consists of two steps. In the first step we compute the so-called example similarity table (EST). It is an $N \times N$ symmetric matrix, where N is the number of examples. All its values are in the range 0.0–1.0. A large similarity value $v_{i,j}$ at position (*i*, *j*), $i\ne j$, denotes a large similarity between examples $ex_i$ and $ex_j$. In the next steps, the EST table is used in the cluster construction process.

#### Step 1: compute example similarity table (EST) through supervised learning

Computation of the example similarity table starts from the original set of *N* examples represented by nominal and numerical attributes that may contain unknown values. An artificial classification problem is defined so that the examples from the original set represent positive examples while negative examples are artificially constructed by shuffling the values of the positive examples. The shuffling is performed at the level of attributes so that we randomly mix values among the examples. The values remain within the same attribute as in the original set of examples. As a result, we have the same attribute values in positive and negative examples, but in negative examples we have randomized connections between the attribute values.

Next, a supervised machine learning algorithm is used to build a predictive model discriminating the positive cases (original examples) and the negative cases (examples with shuffled attribute values). The goal of learning is not the predictive model itself, but the information on the similarity of examples. To this end, appropriate machine learning approaches need to be used, i.e., approaches that are able to determine which examples are classified in the same class in the same manner. For example, in decision tree learning these are the examples which end in the same leaf node, while in decision rule learning these are the examples covered by the same rule. In order to reliably estimate the similarity of examples it is necessary to compute statistics over a sufficiently large set of classifiers (i.e., decision trees or rules). Additionally, a necessary condition for a good estimate is that classifiers are as diverse as possible and that each of them is better than a random classifier. All these conditions are satisfied, for example, by the random forest [17] and random rules algorithms [18]. We use the latter approach in which we typically construct 50,000 rules for each EST estimation.

Similarity between the original (positive) examples is determined as follows. For each pair of the original examples we count how many rules cover both examples. A pair of similar examples will be covered by many rules, while no rules or a very small number of rules will cover pairs that are very different in respect of their attribute values. The exact EST values are obtained by normalization of the above mentioned counts with the largest detected value in the table.

Let us illustrate the EST computation with the following example. Table 1 presents an EST for a subset of 6 examples extracted from a real-world domain [14]. On the left-hand side is the table with numbers of rules covering pairs of examples. Diagonal elements represent total numbers of rules covering each example. After normalization of this table we obtain the EST that is presented on the right-hand side. It can be noticed that we have two very similar examples ($ex_2$ and $ex_5$), three similar examples ($ex_1$, $ex_3$ and $ex_4$), and one very different example ($ex_6$). The maximal value in the table on the left-hand side is 140 and EST values (the table on the right-hand side) are obtained by normalization with this value.

**Table 1:** Example of an EST

	ex$_1$	ex$_2$	ex$_3$	ex$_4$	ex$_5$	ex$_6$		ex$_1$	ex$_2$	ex$_3$	ex$_4$	ex$_5$	ex$_6$
ex$_1$	72	5	51	48	0	19	ex$_1$	0.51	0.04	0.36	0.34	0.00	0.14
ex$_2$	5	140	11	4	118	20	ex$_2$	0.04	1.00	0.08	0.03	0.84	0.14
ex$_3$	51	11	87	62	6	9	ex$_3$	0.36	0.08	0.62	0.44	0.04	0.06
ex$_4$	48	4	62	91	12	7	ex$_4$	0.34	0.03	0.44	0.65	0.09	0.05
ex$_5$	0	118	6	12	125	18	ex$_5$	0.00	0.84	0.04	0.09	0.89	0.13
ex$_6$	19	20	9	7	18	55	ex$_6$	0.14	0.14	0.06	0.05	0.13	0.39

#### Step 2: compute clustering related variability (CRV) score

The second step in the process of clustering uses the example similarity information represented by the EST in order to identify subsets of examples with reduced variability of similarity estimates. For this purpose we define the so-called *clustering related variability (CRV)* score. This is the basic measure used as a heuristic function that guides the search process in the iterative bottom-up clustering algorithm. The CRV score is defined for a single example, but its value depends on the examples it is clustered with, though a cluster may consist of a single example.

The CRV score for an example $ex_i$, denoted by $\mathrm {CRV}_i,$ is the sum of squared deviates of EST values $\{v_{i,j},\ j\in \{1,\dots ,N\}\}$ in row *i* (i.e., example $ex_i$), where $\mathrm {CRV}_i$ is actually computed as a sum of two components:

$$\begin{aligned} \mathrm {CRV}_i = \mathrm {CRV}_{i,wc} + \mathrm {CRV}_{i,oc} \end{aligned}$$

- Within cluster value $\mathrm {CRV}_{i,wc} = \sum _{j\in C}(v_{i,j} - v_{mean,wc})^2$ is computed as a sum over columns *j* of row *i* corresponding to examples included in the same cluster *C* with example $ex_i$. In this expression $v_{mean,wc}$ is the mean value of all $v_{i,j}$ in the cluster. When example $ex_i$ is the only example in cluster *C* then $\mathrm {CRV}_{i,wc}=0$ because we compute the sum only for value $v_{i,i}$ and $v_{mean,wc}=v_{i,i}$.
- Outside cluster value $\mathrm {CRV}_{i,oc} = \sum _{j\notin C}(v_{i,j} - v_{mean,oc})^2$ is defined in the same way as $\mathrm {CRV}_{i,wc}$ but for $v_{i,j}$ values of row *i* not included in cluster *C*. The $v_{mean,oc}$ is the mean value of the EST values in row *i* not included in the cluster and it is different from $v_{mean,wc}$ used to compute $\mathrm {CRV}_{i,wc}$. When example $ex_i$ is the only example in a cluster then $\mathrm {CRV}_{i,oc}$ is the sum of squared deviates for all values in row *i* except $v_{i,i}$.
- Final cluster value $\mathrm {CRV}_{C}$ is computed as the sum of all $\mathrm {CRV}_i$ values of the examples contained in the cluster:
  $$\begin{aligned} \mathrm {CRV}_{C}=\sum _{i\in C} \mathrm {CRV}_i. \end{aligned}$$

#### Step 3: apply CRV score based multi-layer clustering algorithm

Before presenting the multi-layer clustering algorithm used in the experiments, we first describe its single-layer variant.

Based on the CRV score we can define the following bottom–up clustering algorithm that iteratively merges the most similar examples together. In this way it produces a hierarchy of clusters. It should be noted that, in contrast to most other clustering algorithms, this algorithm has a well defined stopping criterion. The process stops when further merging does not result in the reduction of example variability measured by the CRV score. This means that the algorithm automatically determines the optimal number of clusters and that some examples may stay unclustered, or, more precisely, they remain in clusters consisting of a single example. The single-layer clustering algorithm is presented below.
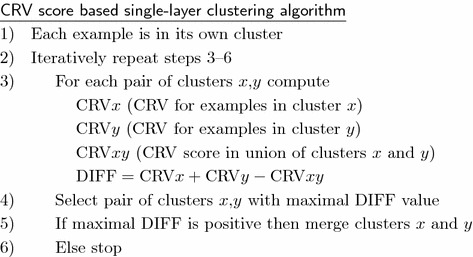


Let us illustrate CRV score and DIFF computation on a simple example. We take the data from the EST presented in Table 1 from which one can notice high similarity between examples $ex_2$ and $ex_5$. We will compute the $\mathrm {CRV}{ex_2}$ value for example $ex_2$ when it is the only example in a cluster, $\mathrm {CRV}{ex_5}$ value for example $ex_5$ when it is in its own cluster, and finally the value $\mathrm {CRV}{ex_2, ex_5}$ when these two examples are together in one cluster. The aim is to demonstrate that DIFF value for this case is positive and large, resulting in a decision to actually put these two examples in one cluster.

1. If example $ex_2$ is the only example in a cluster then $v_{mean,wc}=1.0$ and $v_{mean,oc}=0.23.$
  $$\mathrm {CRV}_{ex_2,wc} = (1.0-1.0)^2=0$$
  $$\mathrm {CRV}_{ex_2,oc} = (0.04-0.23)^2\,+\,(0.08-0.23)^2\,+\,(0.03-0.23)^2\,+\,(0.84-0.23)^2\,+\,(0.14-0.23)^2\,=\,0.484$$
  $$\mathrm {CRV}_{ex_2} = 0.484$$
2. If example $ex_5$ is the only example in a cluster then $v_{mean,wc}=0.89$ and $v_{mean,oc}=0.22$. $\mathrm {CRV}_{ex_5,wc} = (0.89-0.89)^2=0$
  $$\mathrm {CRV}_{ex_5,oc} = (0.00-0.22)^2\,+\,(0.84-0.22)^2\,+\,(0.04-0.22)^2\,+\,(0.09-0.22)^2\,+\,(0.13-0.22)^2\,=\,0.494$$
  $$\mathrm {CRV}_{ex_5} = 0.494$$
3. If example $ex_2$ and $ex_5$ are together in a cluster then for $ex_2$ we have $v_{mean,wc}=0.92$ and $v_{mean,oc}=0.07$. $\mathrm {CRV}_{ex_2,wc} = (1.00-.92)^2+(0.84-0.92)^2=0.012$
  $$\mathrm {CRV}_{ex_2,oc} = (0.04\,-\,0.07)^2\,+\,(0.08\,-\,0.07)^2\,+\,(0.03\,-\,0.07)^2\,+\,(0.14\,-\,0.07)^2\,=\,0.008$$
  $$\mathrm {CRV}_{ex_2} = 0.020$$
4. If example $ex_2$ and $ex_5$ are together in a cluster then for $ex_5$ we have $v_{mean,wc}=0.87$ and $v_{mean,oc}=0.06$. $\mathrm {CRV}_{ex_5,wc} = (0.84-0.87)^2+(0.89-.87)^2=0.001$
  $$\mathrm {CRV}_{ex_5,oc} = (0.00-0.06)^2+ (0.04-0.06)^2+ (0.09-0.06)^2+ (0.13-0.06)^2 =0.009$$
  $$\mathrm {CRV}_{ex_5} = 0.010$$
5. $$\mathrm {CRV}_{ex_2 , ex_5} = 0.030$$
6. DIFF = $$\mathrm {CRV}_{ex_2} + \mathrm {CRV}_{ex_5} - \mathrm {CRV}_{ex_2 , ex_5} = 0.948$$

The basic lesson learned from redescription mining and multi-view clustering is that the reliability of clustering can be significantly improved with a requirement that the result should be confirmed (i.e., difference between CRV scores should be as high as possible) in two or more attribute layers. The approach to clustering based on example similarity presented above for a single-layer case can be easily extended to clustering in multi-layer domains.

If we have more than one attribute layer then we compute the example similarity table independently for each of the layers. For each layer we construct a separate artificial classification problem and construct a separate EST table. Regardless of the number and type of attributes in different layers, EST tables will always be matrices of dimension $N \times N$, because we have the same set of *N* examples in all the layers. After computing the EST tables, multi-layer clustering is performed as described below.


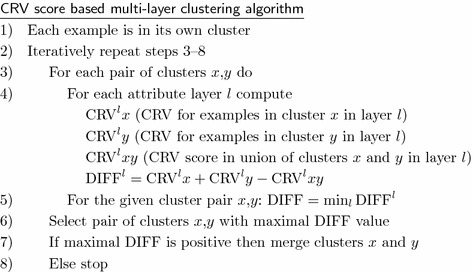
 Conceptually, the multi-layer clustering is identical to the single-layer one. The main difference is that we merge two clusters together only if there is variability reduction in all the layers. For each possible pair of clusters we have to compute potential variability reduction for all attribute layers and to select the smallest value for this pair. If this minimal value is positive it means that merging of the clusters enables variability reduction in all the layers. When there are more pairs with positive minimal value, we chose the pair with the largest minimal value and then we merge these clusters in the current iteration. When we perform clustering in two or more layers we have a conjunction of necessary conditions for merging two clusters. A consequence is that resulting clusters are smaller than in the case of a single-layer clustering.

Positive properties of the approach are that no explicit distance measure is used, that the algorithm has a well-defined stopping criterion, that examples can include both numerical and categorical attributes with unknown values, and that availability of many attributes is an advantage. The algorithm cannot be used in domains with a very small number of attributes and in applications like text mining in which attribute values are sparse. The main disadvantage of the approach is high sensitivity to the existence of attribute copies. The multi-layer concept can be regarded as a way to ensure attribute diversity at least at the level of different layers. Finally, it must be noted that the final result of the algorithm is a potentially large set of clusters and that some of clusters may contain only a small number of examples. Which clusters will be selected for the expert evaluation is the task for a human evaluator. A common practice is to select a small number of large clusters. In this work we have selected three largest clusters.

The algorithm is publicly available as a web service at http://www.rr.irb.hr/MLC/index.php. The instructions as well as an illustrative data set that may be used for experiments are also available.

## Results

By applying the multi-layer clustering to the available data set we identified three clusters of patients with significant problems with dementia. Table 2 presents the basic information about the clusters: total number of included patients, number of patients with AD, LMCI, and EMCI diagnosis that are in the cluster, and mean value of the CDRSB score for included patients. In the last column are the names of clusters that have been given after the analysis of the properties of included patients.

**Table 2:** Three clusters of patients with significant dementia problems. In the last column are the names of clusters given according to the properties of the included patients

Cluster	Number ofpatients	Patients with diagnoses			CDRSB score	Description
		AD	LMCI	EMCI		
A	35	30	4	1	4.81	Severe atrophy AD
B	21	10	9	2	3.55	Mild self-critical AD
C	42	30	10	2	4.15	Traumatic AD

The first cluster (A) includes patients with very small volumes of all relevant parts of the brain: hippocamus, entorhinal, fusiform gyrus, and middle temporal gyrus. Small are also intracerebral volume and the volume of the whole brain. From Table 3 it can be seen that for fusiform and midtemp the mean values for patients in the first cluster are about 20 % lower than the mean values for the cognitive normal ADNI population. For Entorhinal volume the difference is about 30 %. Atrophy for patients in this cluster is so high that their mean volumetric data are lower than the mean value for all 148 patients with AD diagnosis. The clinical properties of patients in this cluster are characteristic for patients with severe problems with dementia: very high CDRSB, ADAS13, and FAQ scores and very low MMSE and MOCA scores (Table 4). Table 4 also presents FDG-PET values that are in accordance with the status of patients in this cluster. The name “severe atrophy AD” that is given to the cluster denotes patients that have severe problems with dementia as a consequence of a strong degenerative process. The cluster consists of about 65 % of female patients. The cluster includes 30 patients with AD diagnosis, four patients with LMCI diagnosis, and one patient with EMCI diagnosis (see Table 2). The question why these patients with LMCI and even EMCI diagnosis have been included into a cluster with severe problems with dementia deserves special attention but is out of the scope of this work. ADNI identification numbers for these patients are 2194 (EMCI), 4034, 4167, 4430, and 4502 (LMCI).

**Table 3:** Mean values of MRI volumetric data for clusters A, B and C

Cluster	Hippocampus	Entorhinal	Fusiform	MidTemp	ICV	Wholebrain
A	5.52	2.7	14.5	15.3	1388	912
B	6.05	3.1	16.7	18.7	1464	1011
C	6.46	3.3	17.8	19.3	1710	1124
AD	5.91	2.9	16.3	17.8	1509	1006
CN	7.53	3.9	18.7	20.8	1487	1054

Actual values are reduced by 1000. In last two rows are the mean values for 148 patients with AD diagnosis and 187 cognitive normal patients

**Table 4:** Mean values of FDG-PET data and four clinical scores for clusters A, B and C

Cluster	FDG	ADAS13	MMSE	MOCA	FAQ
A	4.72	35	22	15	15
B	5.68	24	25	20	10
C	5.07	30	24	18	12
AD	5.34	31	23	17	13
CN	6.59	9	29	26	0

The second cluster (B) is named “mild self-critical AD.” It includes 10 patients with AD, nine patients with LMCI, and two patients with EMCI diagnosis. The patients are characterized with moderately decreased volumes that are about 20 % for Entorhinal and Hippocampus, and 10 % for Fusiform and midtemp lower than mean values for cognitive normal patients. The intracerebral volume and whole brain volume are about normal values. Clinical picture is typical for AD patients (CDRSB score above three) but values for all scales demonstrate a mild form of AD. A decisive property of patients in this cluster is that for all cognitive functions problems values reported by patients themselves are higher than for other clusters and higher than the mean value for the complete AD population (see Table 5). The result suggests self-criticism of included patients. This is an interesting observation because self-criticism is not characteristic for patients that have problems with dementia and typically it is less expressed as the disease progresses. The cluster includes about 60 % of female patients.

**Table 5:** Mean values of cognitive functions questionnaire demonstrating self-criticism filled out by patients in cluster B

Cluster	EcogPt						
	Memory	Language	Visuosp.	Planning	Organization	Div. att.	Total
A	2.35	1.81	1.55	1.40	1.59	1.90	1.81
B	2.60	1.95	1.81	1.62	1.70	1.98	1.97
C	2.29	1.78	1.49	1.50	1.50	1.61	1.77
AD	2.40	1.88	1.60	1.62	1.74	1.91	1.89
CN	1.54	1.35	1.15	1.12	1.25	1.42	1.32

The third cluster (C) consists of patients with the least expressed degenerative changes in respect of hippocampus, entorhinal, fusiform, and midtemp (see Table 3) but which are accompanied by *high* values of the ventricular volume, the intracerebral volume, and the whole brain volume (see Table 6). Mean value for the ventricles is about 100 % higher than for cognitive normal patients. Patients in clusters A and B also have increased ventricles but their size is significantly smaller. It is very important to notice that in spite of the low level of brain atrophy the included patients have unexpectedly expressed problems with dementia, and for all scales, including the FDG-PET, the values are between the values of the first and the second cluster (see Table 4). Clinically the patients in this cluster have specifically problems with RAVLT forgetting and divided attention as reported by the patient study partner (see Table 6). We name this cluster “traumatic AD” because it is known that head injuries may cause ventricular enlargement [19]. The supporting fact is that 85 % of included patients are males and brain injuries in some male sports like boxing and football as well injuries during military service may result in significant problems with dementia [20, 21]. Data available in the ADNI database did not allow us to evaluate this hypothesis.

**Table 6:** Characteristics of patients in cluster C

Cluster	Ventricles	ICV	Wholebrain	RAVLT forgetting	EcogSP Div att.
A	43.1	1388	912	4.20	2.86
B	41.4	1464	1011	4.76	2.85
C	64.9	1710	1124	4.86	2.94
AD	47.8	1509	1006	4.42	2.90
CN	32.5	1487	1054	3.80	1.25

**Fig. 1:**
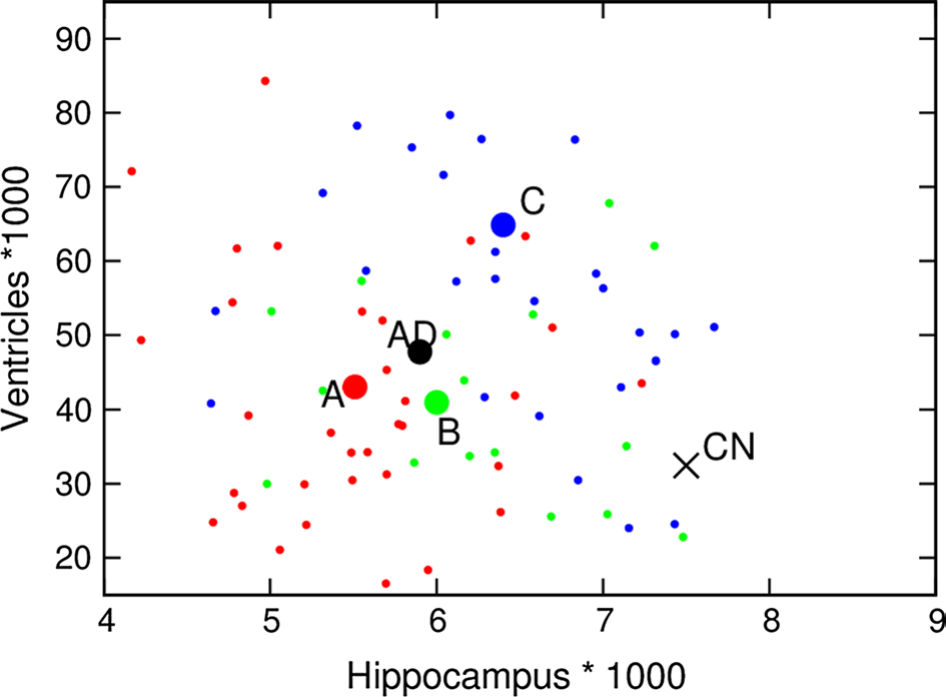
Ventricles versus hippocampus volumes. Distribution of patients in cluster A (*red circles*), cluster B (*green circles*), and cluster C (*blue circles*) is presented by their hippocampus and ventricles volume values. * Big circles* present mean values of clusters. The big “$\times$” represents the mean value for 187 CN patients while the* big black circle* represents the mean value for AD patients

The relation between brain degeneration and ventricular enlargement for clusters A, B and C is presented in Fig. 1. Hippocampal volume is selected for the illustration of the brain degeneration. Small red points represent patients in cluster A, small green points are patients in cluster B, while patients in cluster C are denoted by blue points. Large circles denote mean values in corresponding clusters. The position of the black “$\times$” denotes the mean value for 187 cognitive normal patients while the big black circle is the mean value for 148 patients with AD diagnosis. At first we can notice a significant difference between cognitive normal and AD patients both in respect of hippocampal volume and ventricular volume. But even more interesting are the differences among clusters. Hippocampal atrophy (and also entorhinal, fusiform, and midtemp volumes) is most severe for cluster A and least expressed for cluster C. In contrast, ventricular enlargement of similar intensity is present for clusters A and B while it is especially expressed for cluster C.

**Fig. 2:**
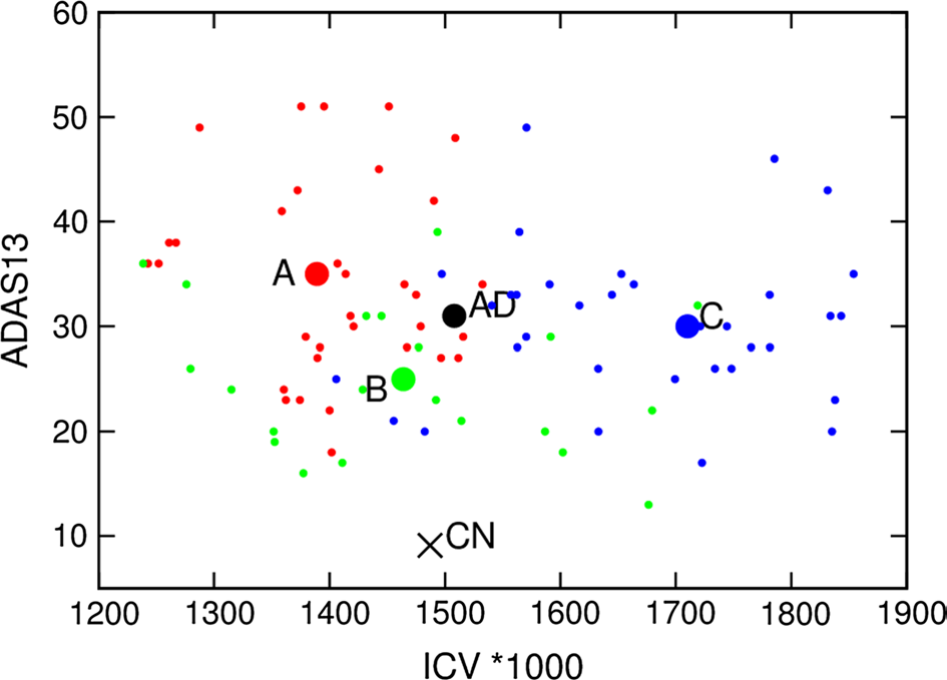
ADAS14 score versus intracerebral volume.Relation between ADAS13 score and ICV values for patients in cluster A (*red circles*), cluster B (*green circles*), and cluster C (*blue circles*).* Big circles* present the mean values of clusters. The big “$\times$” represents the mean value for 187 CN patients while the* big black circle* represents the mean value for AD patients

Figure 2 illustrates relations between values of ICV and clinical severity of dementia. ADAS13 score is used as a measure of the clinical status of patients but similar properties are present also for CDRSB, FAQ, MOCA and MMSE. At first it should be noted that in respect of ICV there is no significant differences between cognitive normal patients and AD patients when we compare mean values of the complete populations but that there are significant differences when we look at specific patients and mean values of clusters. Patients in cluster C (blue points) are mostly in the right-hand part of the figure while red and green points (patients in clusters A and B, respectively) are in the left-hand part. Patients in all three clusters have significant problems with dementia (ADAS13 values above 20), clinical symptoms are very expressed for clusters A and C while they can be characterized as mild for the majority of patients in cluster B.

## Discussion

The results demonstrate that the population of patients that have problems with dementia is very non-homogeneous and that population of patients diagnosed as cognitive normal is even less homogeneous than the population of patients with AD diagnosis. This can be concluded from the fact that constructed clusters are small and that all three largest clusters are subgroups of patients in which those with AD diagnosis are the majority. The total number of patients with AD diagnosis included into clusters A, B and C is less than 50 % of all AD patients. This means that our clusters should be interpreted as the most homogeneous subpopulations of AD patients and not as a segmentation of the complete AD population.

Very elaborate medical studies have recently shown that mild and severe cognitive impairment as well as AD diagnosis are correlated with atrophy of the brain. Our results are completely in agreement with these findings: degenerative changes of the brain are present in all three identified clusters. The strongest atrophy is characteristic for patients in cluster A and it is accompanied by the most severe clinical properties of patients. Significant atrophy is present in cluster B and although it is less expressed in cluster C, lower than normal volumes of hippocamus, entorhinal, fusiform gyrus, and middle temporal gyrus can be encountered practically in all included patients. The novelty of our result is the identification of cluster B which includes patients that demonstrate a property of self-criticism, and especially the identification of cluster C for which it may be assumed that medical problems are a consequence of previous brain injuries.

The relevance of cluster C is manifold. First, it is interesting that this is the largest identified cluster. This is not a proof that injuries are the most common cause of problems with dementia because only about 20 % of patients with AD diagnosis included into the analysis are actually present in cluster C. But the result suggests that a significant part of problems with dementia can be attributed to previous brain injuries. The properties of patients in cluster C suggest that brain injuries alone are not enough for the development of AD: it is necessary that they are accompanied by at least mild brain atrophy. It is perhaps the reason that consequences of brain injuries are not present immediately after the injury but only after many years when due to aging atrophy naturally happens.

Our results demonstrate that large volume of ventricles that can be attributed to injuries of patients included into cluster C is unexpectedly correlated with large ICV and the whole brain volumes. The result is in agreement with our previous results [15, 16] which demonstrated existence of male AD subpopulations with such properties. Although differences among clusters for ICV values are less expressed than differences for ventricles, differences for ICV and whole brain volumes are statistically more significant than differences for ventricles because the direction of differences is opposite: due to atrophy ICV and whole brain volumes are decreased while due to injures they are increased. It can be observed that a significant number of cognitive normal, especially male patients in the ADNI database also have increased values of ICV. An important issue is whether these patients also have a history of previous brain injuries and if they will develop AD when even a slight brain atrophy will take place.

Finally, it is interesting to notice that the mean values of FDG-PET presented in Table 4 for clusters A, B and C decrease proportionally to the severity of the disease regardless of the causes of the disease and the way volumetric values change. The result is not surprising because it is in accordance with previously published results [3]. A lesson learnt from our work is that FDG-PET is not useful for understanding the underlying biological processes but it is the most useful among available biological descriptors for the objective evaluation of the clinical status of patients.

## Conclusions

This work presents the application of a novel clustering methodology for identification of small but homogeneous subsets of patients with significant dementia problems. The methodology is based on a novel concept of co-existing properties as a main motivation for building clusters. The evaluation of the properties of constructed clusters has clearly demonstrated usefulness of the developed algorithm. But the methodology has also some deficiencies, among which sensitivity to inclusion of highly correlated attributes is the most significant. Because of that the implemented algorithm cannot be recommended as a general purpose clustering tool. The success in the AD domain is mainly due to the availability of many attributes distributed in two different data layers. It may be expected that the methodology will be successful also in other similar medical and non-medical domains.

AD domain is a good example of a task in which relations between layers can be different for various subpopulations. In such cases it is very difficult to identify relevant relations on a complete population by classical statistical analysis and supervised machine learning approaches. But after successful identification of homogeneous subpopulations even a simple analysis of mean values and standard deviations may enable discovery of relevant relations.

A drawback of the methodology is that constructed clusters are small and that they tend to be even smaller if more than two layers are used. In the concrete AD domain we got some useful insights about less than a half of AD patients and practically no insight about cognitive normal patients and patients with mild impairment. Additionally, the methodology has a high computational complexity, which is growing quadratically with the number of examples. Because of this the methodology is currently not applicable to domains with more than a few thousands of examples.
